# Molecular characterization of Orf virus in goats in Gabon, Central Africa

**DOI:** 10.1186/s12985-016-0535-1

**Published:** 2016-05-13

**Authors:** Gael D. Maganga, Anthony Relmy, Labib Bakkali-Kassimi, Barthélémy Ngoubangoye, Thierry Tsoumbou, Christiane Bouchier, Nadine N’Dilimabaka, Eric M. Leroy, Stéphan Zientara, Nicolas Berthet

**Affiliations:** Centre International de Recherches Médicales de Franceville (CIRMF), BP769, Franceville, Gabon; Institut National Supérieur d’Agronomie et de Biotechnologies (INSAB), Franceville, Gabon; French Agency for Food, Environmental and Occupational Health and Safety (ANSES), Maisons-Alfort, France; Institut Pasteur, Plate-forme Génomique - Pôle Biomics, 28 rue du Dr. Roux, 75724 Paris, France; Institut de Recherche pour le Développement (IRD), Maladies Infectieuses et vecteurs: Ecologie, génétique, Evolution et Contrôle (IRD 224 – CNRS 5290 6 UM1- UM2), Montpellier, France; Centre National de la Recherche Scientifique (CNRS), UMR3569, 25 rue du docteur Roux, 75724 Paris, France

**Keywords:** Orf virus, Goats, High-throughput sequencing, PCR

## Abstract

**Background:**

Orf or contagious ecthyma is a zoonotic viral infection with a potential serious health threat for the small ruminants industry as well as humans. It is currently emerging in new territories.

**Results:**

Eight suspected clinical cases of pustular dermatitis in goats occurred in the rural area of Tebe, in south-eastern Gabon, in January 2013. The orf virus (ORFV) was detected by high-throughput sequencing on sera, buccal swabs and scab pool samples. It was confirmed in six out of eight sick goats by using specific PCR targeting the major envelope protein (B2L) and the orf virus interferon resistance (VIR) genes. Phylogenetic analysis revealed that the Gabonese strain and South Korean strains evolved from a common ancestor, suggesting an Asian origin of the ORFV’ Gabonese strain.

**Conclusions:**

This study provides the molecular detection of the ORFV strain involved in the cases of pustular dermatitis in goats and highlights its circulation in Gabon.

## Background

Orf or contagious ecthyma is a zoonotic viral infection caused by an enveloped double stranded DNA virus, the orf virus (ORFV) belonging to the *Poxviridae* family, *Chordopoxvirinae* subfamily, *Parapoxvirus* genus. This infectious pustular dermatitis primarily affects sheep, goats and wild ruminants worldwide [1]. Orf is characterized by proliferative skin lesions of the lips, muzzle, ears, eyelids, and found around the mouth and nostrils of lambs [2]. The disease also results in genital, udder and foot lesions. Additional lesions are observed in the oral mucosa in severe forms of disease. The transmission within a herd is carried out through direct contact between animals during confrontation or suckling. The morbidity can approach 100 %, whereas the mortality is usually less than 1 %. Moreover, humans (farmers, butchers, sheep and goats shearers and veterinarians) can also be infected by direct contact with sick animals. The preferential location of the lesions in humans is the hand. A rash-shaped papule can be seen extending progressively and forms a pustule in its center. After a few days, the lesion evolves and can become very voluminous. The lesion can present nodules or patches which can be very oedematous, even vesicular. An associated satellite lymphangitis or adenopathy can be observed. Some cases accompanied by fever have been described [3]. Consequently, orf has become a potential serious health threat for the small ruminants industry with an important economic impact as well as for humans.

The importance of orf infection has recently increased due to the emergence of this virus in new territories, the occurrence of re-infection of previously infected animals, as well as interspecies infection [4]. Several studies seem to indicate a very high incidence of orf infection (75 %) during the dry season in the central African region, although actual prevalence is still greatly underestimated within livestock herds and in humans [4, 5]. In Africa, orf infection has been reported in sheep and goats only in a few countries such as Cameroon, Nigeria and Tanzania [5], on the basis of clinical signs, and in camels in Kenya, Somalia and Sudan [6–8]. To date on the African continent, the ORFV was only detected by a molecular diagnostic assay from goats and sheep in Ethiopia, Egypt, and South Africa where the disease is endemic [9, 10, 11]. In Gabon, no case had yet been recorded. In this study, we report the first cases of orf infection causing a small outbreak of pustular dermatitis in goats in south-eastern Gabon and the molecular characterization of the viral strain.

## Methods

### Clinical and epidemiological investigations

**Fig. 1:**
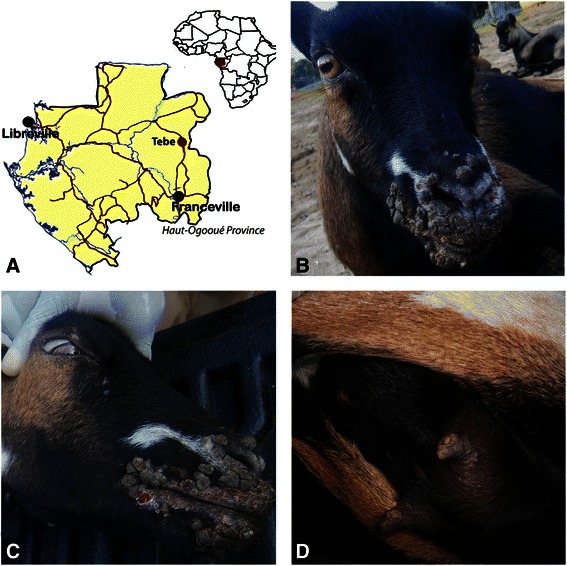
Geographic location and typical clinical signs of the ORFV infection in goats. Expanded map of Gabon showing the geographic location of confirmed cases of orf infection among goats, south-eastern Gabon (red circle) (**a**), proliferative lesions of ORFV infection around the lips and nostrils of goats (**b**, **c**), proliferative lesions on the udders (**d**)

In January, in the rural area of Tebe, located in the Province of Haut-Ogooué in south-eastern Gabon (Fig. 1a), eight suspected clinical cases of pustular dermatitis were reported. Clinical examination of the goats and the sampling process were carried out by a field veterinarian.

### Sample collection

Approval was given by the local administrative authorities to carry out the sampling and the diagnosis of the causative agent of the disease observed in the goats of the Tebe Village. All clinical samples, including plasmas, sera, as well as buccal and ocular swabs and buccal scabs were collected from sick animals following the recommendations of the OIE Terrestrial Manual and stored at -80 °C and sent to the laboratory of the Centre International de Recherches Médicales de Franceville (CIRMF, Gabon) for diagnosis.

### Serological and virological screening

According to their non-specific nature, these symptoms can be common to several vesicular diseases of sheep and goats such as foot and mouth disease and bluetongue disease [12], both listed by the World Organisation for Animal Health as high-impact diseases. The two viruses causing the aforementioned diseases were initially searched using specific tools. Indeed, plasmas and sera were first tested by enzyme-linked immunosorbent assays (ELISA) to look for the presence of foot and mouth disease virus (FMDV) and bluetongue virus (BTV) antibodies as previously described [13, 14]. In parallel, total viral RNA was extracted from all clinical samples using the EZ1 Virus Mini Kit version 2.0 according to the manufacturer’s instructions, and BTV [15] and FMDV [16] specific real-time reverse transcription PCR assays were carried out. Viral isolation was attempted from buccal scabs and sera using Vero cells.

### Molecular characterization of ORFV by high-throughput sequencing

An approach based on the high-throughput sequencing of RNA extracted from three selected pools (one pool of 8 sera, one of 6 buccal swabs and the last of 4 buccal scabs) was performed. RNA was extracted using QIAmp Viral RNA Minikit (Qiagen), treated with DNAse to remove contaminating DNA, and then retro-transcribed using SuperScript III enzyme and random hexamers (Invitrogen Inc., Carlsbad, CA). Amplification was done by using Phi29 enzyme. The Illumina Sequencing using HiSeq 2000 was conducted with a mean depth per sample of 20 x 10^6^ single reads of 100 nucleotides (nt) size. After removal of low-quality reads, a taxonomic assignment was performed by BLASTN against the EMBL database on each read for each selected pool.

### ORFV specific PCR amplifications

In order to confirm the identification of the ORFV as causative agent of these lesions, an ORFV-specific real-time PCR assay targeting the B2L gene was performed on buccal swabs and buccal scabs as previously described [17]. Then, two specific conventional PCR targeting the major envelope protein (B2L) and the orf virus interferon resistance (VIR) genes were performed, as described previously, on all real-time PCR positive samples [18].

### Phylogenetic analyses

B2L and partial VIR gene sequences of Gabonese ORFV strains were aligned with homologous sequences of parapoxvirus reference strains from GenBank, using the ClustalW algorithm of MEGA program version 5 [19]. Bayesian inference of phylogeny was done using the MrBayes V.3.2 software and the GTR + G + I nucleotide substitution model [20] for two million generations with a burn-in of 25 %.

## Results

Several flocks of goats are present in the rural area of Tebe. Among the herd of about fifty goats on which the sampling was performed, eight suspected clinical cases of pustular dermatitis were only reported in this area during two weeks in January 2013. No mortality was observed. Relevant clinical signs (Table 1) such as buccal pustules, lip scabs, proliferative and ulcerative lesions on the nostrils (Fig. 1b and c) and proliferative lesions on the udders and vulva (Fig. 1d) were observed.

**Table 1:** Clinical data and results of the serological and virological investigations

Id animal	Sex	Clinical signs	FMDV		BTV		ORFV	
			ELISA	qRT-PCR	ELISA	qRT-PCR	B2L PCR	VIR PCR
1	F	Proliferative lesions around the mouth, pale mucosae, inflammation of the retro-mandibular lymph nodes, hyperthermia (39,7 °C)	Neg	Neg	Neg	Neg	Neg	Neg
2	F	Abscess at the trough, proliferative lesion around the lips, lacrimation, conjunctivitis, lymphadenitis poly, hyperthermia (40,5 °C)	Neg	Neg	Neg	Neg	Neg	Neg
3	F	Inflammation of the retro-mandibular lymph nodes, diarrhea, lip scabs, hyperthermia (39.8 °C)	Neg	Neg	Pos	Neg	Neg	Pos
4	F	Inflammation of the retro-mandibular lymph nodes, proliferative lesions around the lips and nostrils, hyperthermia (40 °C)	Neg	Neg	Neg	Neg	Neg	Pos
5	M	Ulcerative lesions on the nostrils, hyperthermia (39.9 °C)	Neg	Neg	Neg	Neg	Pos	Pos
6	F	Buccal scabs, proliferative lesions in the mouth, on udders and vulva, hyperthermia (40.3 °C)	Neg	Neg	Neg	Neg	Pos	Pos
7	F	Proliferative lesions on the lips, buccal scabs, hyperthermia (40.1 °C)	Pos	Neg	Pos	Neg	Neg	Pos
8	F	Proliferative lesions on the lips and udders, conjunctivitis, lacrimation, hyperthermia (39.7 °C)	Neg	Neg	Neg	Neg	Pos	Pos

Out of eight sera samples, only two were found positive for BTV antibodies, and one of the two individuals was also found positive to FMDV as well (Table 1). Subsequently, specific BTV and FMDV real-time reverse transcription PCR assays yielded no positive results for all the clinical samples tested. Moreover, viral isolation attempted from buccal scabs and sera from six animals (cases 3, 4, 5, 6, 7 and 8) showed no cytopathic effect.

Sera, buccal swabs and scab pools samples selected for high-throughput sequencing yielded a total of 376,700, 380,629 and 127,723 reads, respectively, corresponding to the viral sequences of the ORFV. Both buccal swabs and buccal scabs were positive for ORFV-specific RT-PCR. For buccal swabs, Ct values ranged from 24.7 to 37.82. For buccal scabs Ct values were 21.44 and 31.22. The highest viral load was found in buccal scabs. Subsequently, the ORFV was detected by specific PCR in six out of eight goats (Table 1). The sequencing of amplified PCR products enabled us to obtain two fragments of 546 and 1077 bp for the partial VIR and B2L genes, respectively.

**Fig. 2:**
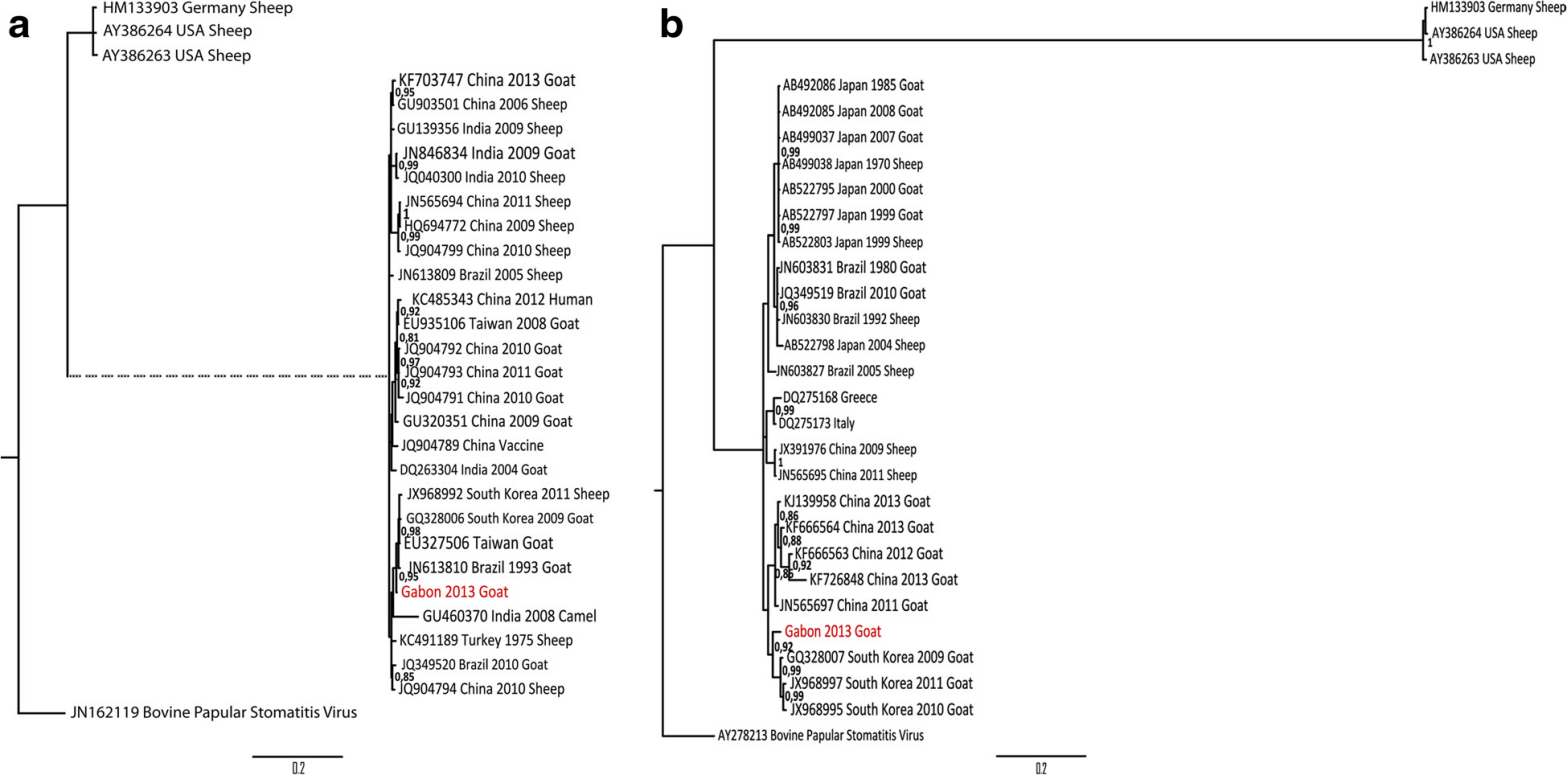
Phylogenetic tree based on nucleotide sequences of the B2L gene (**a**) and VIR gene (**b**) analysis. The tree was visualized with FigTree 1.3.1. Bayesian posterior probabilities values are shown to the right of the branches. ORFV sequences obtained in this study are shown in red. In Fig. 2a, a branch has been shortened and condensed for clarity and represented as dashed branch

Based on analysis of the sequences of the B2L gene, the Gabonese ORFV strain displayed 99 % of identity at the nucleotide level with the South Korean (JX968992 and GQ328006), Brazilian (JN613810) and Chinese (JQ904794) strains. As before, analysis of the sequences of the partial VIR gene indicated that the Gabonese ORFV strain displayed 97 % of identity at nucleotide level with the South Korean strains identified in 2009 (GQ328007), 2010 (JX968995) and 2011 (JX968997) and the Chinese strain (JN565697). Phylogenetic analysis based on the partial B2L (Fig. 2a) and VIR (Fig. 2b) genes indicated that the Gabonese strain was closely related to the Asian strains, especially the South Korean strains, with which it has evolved from a common ancestor (Fig. 2b).

## Discussion

We provide, in this study, the first clinical description and genetic characterization of the orf infection involved in an outbreak in goats from a rural area in south-eastern Gabon. No case had been previously recorded in Gabon in spite of various orf infection already reported in sheep and goats in other countries of Central Africa such as Cameroon and Nigeria [5]. However, given the ubiquitous nature of this virus in small ruminant populations worldwide (and in proximate regions to Gabon) and the fact that most infections are subclinical, it is unlikely this diagnosed case of clinical orf represents the first appearance of ORFV in Gabon. The comparative sequence analysis revealed that the Gabonese ORFV strain displayed 99 % of identity at nucleotide level with the Asian strains, especially the South Korean strains. These findings would suggest a potential introduction of this Gabonese ORFV strain from Asian countries, possibly through their economic activities in Central Africa. This orf infection does not seem to be the first case of a virus importation in Africa from Asia. The recent emergence of the Asian “*Peste des Petits Ruminants”* Virus lineage IV isolated in Central Africa would be a good example of the importation of a virus from this continent [21]. The presence of the disease in Cameroon could otherwise have led to an introduction from this neighboring country as had been suggested for the “*Peste des Petits Ruminants*” [22]. However, no concrete evidence to support this hypothesis is available.

In rural areas of Gabon, small ruminants are bred by straying and frequent contacts between humans and lambs or kids, which are highly susceptible to orf infections, could lead to human infection, particularly for people who butcher these animals. A serological survey would be needed to assess the level of virus circulation in animals and humans in order to find out its impact on livestock, which compose one of the main sources of animal proteins in villages totally devoid of commercial shops. Thus, a prospective program should be implemented to control, and eradicate in the future, orf infection in Gabon by vaccinating small ruminants in order to limit the spread of the disease. Further molecular and serological analysis could be needed to better understand the circulation of the ORFV strains between humans and domestic animals.

In addition to the recent identification of the ORFV in Gabon, another virus belonging to the *Poxviridae* family, the monkeypox virus, has also been identified in Gabon [23] and in other Central African countries among which the Central African Republic [24], the Democratic Republic of the Congo [25] and recently Cameroon (according to the notification by the Cameroonian veterinary service at the World Organisation for Animal Health on July 18th, 2014). The identification of these two viruses highlight the potential emergence of poxviruses and the risk of spread of the ORFV in Central Africa.
